# Obscure gastrointestinal bleeding caused by small intestinal lipoma: a case report

**DOI:** 10.1186/s13256-016-1014-4

**Published:** 2016-08-12

**Authors:** Noboru Yatagai, Hiroya Ueyama, Tomoyoshi Shibuya, Keiichi Haga, Masahito Takahashi, Osamu Nomura, Naoto Sakamoto, Taro Osada, Takashi Yao, Sumio Watanabe

**Affiliations:** 1 Department of Gastroenterology, Juntendo University, School of Medicine, 2-1-1 Hongo, Bunkyo-Ku Tokyo, 113-8421 Japan; 2 Department of Human Pathology, Juntendo University, School of Medicine, Tokyo, Japan

**Keywords:** Obscure gastrointestinal bleeding, Lipoma, Small intestine, Double-balloon endoscopy, Capsule endoscopy, Case report

## Abstract

**Background:**

Small intestinal lipomas are rare, usually asymptomatic, and most commonly encountered incidentally during investigation of the gastrointestinal tract for another reason. However, they may cause obscure gastrointestinal bleeding.

**Case presentation:**

We report a case of obscure gastrointestinal bleeding due to a small intestinal lipoma. A 69-year-old Japanese man on antiplatelet therapy presented to our department with tarry stools and anemic symptoms. A small intestinal tumor was detected by capsule endoscopy and double-balloon endoscopy. After laparoscopic resection, the tumor was confirmed to be a lipoma.

**Conclusions:**

Small intestinal lipomas are difficult to detect by conventional modalities, but capsule endoscopy and double-balloon endoscopy are good modalities for the diagnosis of small intestinal lipomas. Treatment of small intestinal lipomas should be selected carefully, considering the tumor size, size of stalk, administration of antithrombotic therapy, and endoscopic operability.

## Background

Obscure gastrointestinal bleeding (OGIB) is a very rare entity that accounts for less than 5 % of all cases of gastrointestinal (GI) bleeding [1]. Small bowel tumors are an infrequent but serious cause of OGIB [2]. GI lipomas are benign, usually single, slow-growing, non-epithelial tumors. The most common site is the colon, although they may also be found in the stomach, esophagus, and small intestine [3]. In general, lipomas are larger than 2 cm in diameter, and tend to produce symptoms such as GI bleeding, anemia, intussusception, and bowel obstruction [3]. Zhang *et al.* [4] reported that the common causes of small intestinal bleeding were: vascular anomalies (54.35 %), small intestinal ulcer (13.04 %), and small intestinal tumors (11.96 %) in older patients (>65 years); vascular anomalies (34.82 %), small intestinal tumors (31.25 %), nonspecific enteritis (9.82 %) in middle age (41–64 years); Crohn’s disease (34.55 %), small intestinal tumors (23.64 %), and nonspecific enteritis (10.91 %) in young adults (<40 years) [4, 5].

We describe a case of OGIB due to a small intestinal tumor that was preoperatively diagnosed by capsule endoscopy (CE) and double-balloon endoscopy (DBE). The patient subsequently underwent laparoscopic surgery because endoscopic treatment may have caused hemorrhage or perforation. Pathologic analysis confirmed that the small intestinal tumor was a lipoma.

## Case presentation

A 69-year-old Japanese man presented to our department with tarry semiliquid stools and symptoms of anemia. He had a medical history of hypertension, hyperlipidemia, and type 2 diabetes treated with oral and insulin therapy. Furthermore, he was receiving antiplatelet therapy (clopidogrel 75 mg/day) for a cerebral infarction that had occurred 2 months previously. On physical examination, he was pale, with a temperature of 36.0 °C, a pulse rate of 85 beats per minute, a blood pressure of 107/63 mmHg, and a respiration rate of 14 breaths per minute. A rectal examination revealed tarry fecal content. Laboratory tests were indicative of normocytic-normochromic anemia (hemoglobin 12.4 g/dL, normal 13.4 to 17.1 g/dL). The results of all other laboratory tests were unremarkable.

**Fig. 1:**
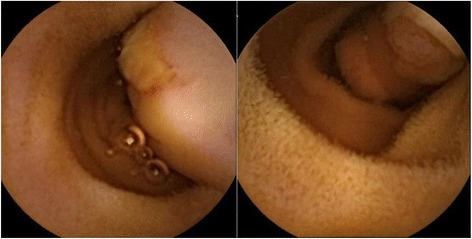
Capsule endoscopy showing a pedunculated submucosal tumor with an ulcer in the upper jejunum

**Fig. 2:**
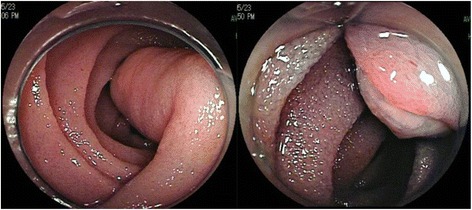
Double-balloon endoscopy showing a cylindrical submucosal tumor with an ulcer in the upper jejunum

**Fig. 3:**
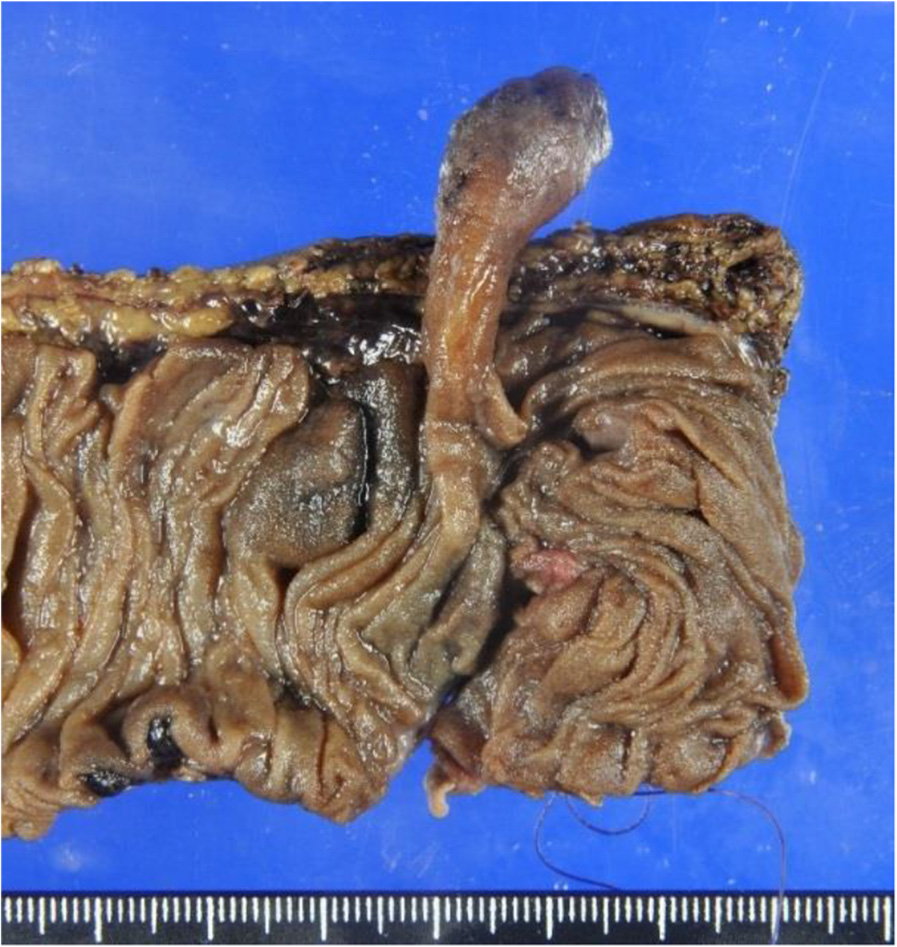
The resected specimen showing a pedunculated tumor

**Fig. 4:**
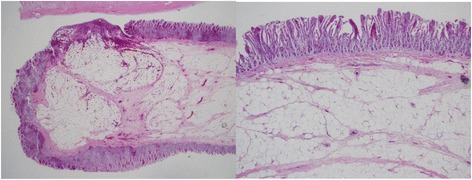
Microscopic examination of the resected tumor showing a lipoma consisting of mature fat cells with an ulcerated surface (hematoxylin and eosin staining)

After he was stabilized, an extensive diagnostic workup was initiated. No evidence of bleeding was detected by esophagogastroduodenoscopy (EGD) and colonoscopy (CS). Abdominal contrast-enhanced computed tomography (CT) scans and CT angiography did not reveal the cause of bleeding. Therefore, he underwent CE for assessment of his small intestine. CE showed a pedunculated submucosal tumor with an ulcer in his upper jejunum (Fig. 1). Subsequently, DBE via an oral route revealed an ulcerated submucosal tumor, 35 mm in size, which was in the same location (150 cm distal from the ligament of Treitz) as that found on CE (Fig. 2). The tumor was cushion-sign positive. We speculated that it was an inflammatory fibroid polyp because it had a pedunculated cylindrical lesion with an ulcer on the top of the lesion. A biopsy and endoscopic treatment were not performed due to the large size and wide stalk of the lesion, which were considered to increase the possibility of operational defects, and the risk of active bleeding and perforation. Black ink injection and clips were used to mark the anal side of the lesion. Our patient underwent laparoscopic surgery of his small intestine (segmental resection; Fig. 3). A pathological examination of the resected tumor showed a pedunculated lipoma (36×9 mm) of the jejunum, consisting of mature fat cells with an ulcer (Fig. 4). His postoperative course was uneventful and he was discharged 9 days later. He remained well at 13 months after surgery, without evidence of recurrent bleeding.

## Discussion

GI lipomas are benign tumors of mesenchymal origin, which are usually single and slow growing [3]. Their occurrence is most common in the colon, but they can also be found in the small intestine, and very rarely in the esophagus and stomach [6]. Primary small intestinal tumors are rare and account for approximately 1 % of all GI tumors [7]. Primary lipomas of the small intestine are also unusual, representing 2.6 % of nonmalignant tumors of the intestinal tract [8]. The most common site in the small intestine is the ileum, followed by the jejunum [3].

On pathological examination, GI lipomas are composed of a spherical deposit of adipose tissue in the wall of the GI tract, usually in the submucosal layer. Because of peristalsis, extrusion of the lipoma into the lumen of the GI tract is repeated, which may cause the formation of pseudopedicles [6].

On endoscopic examination, lipomas tend to appear as yellowish, smooth, round or hemispherical tumors, with either a pedunculated or wide stalk [9]. The cushion sign and naked fat sign are also specific to lipomas [9]. Sawada *et al.* [10] and Chou *et al.* [9] reported that small intestinal lipomas causing OGIB were characterized by a wide stalk and ulceration. In contrast, in the present case, the small intestinal tumor was whitish, pedunculated, and cylindrical, with ulceration on the top. Endoscopic investigation did not allow conclusive diagnosis of a typical small intestinal lipoma. Endoscopists should be aware of small intestinal lipomas that exhibit a whitish color and cylindrical shape.

Preoperative diagnosis of small intestinal lipomas is difficult without CE and DBE. On abdominal CT scans, lipomas appear as round, smooth, well-demarcated tumors with a fat attenuation coefficient of –80 to –120 Hounsfield units, when the tumor size is large [11]. In the present case, a lipoma was not identified because the tumor was pedunculated and cylindrical. In small intestinal series, lipomas may appear round in shape. Small intestinal series are effective for detection of the tumor site, and can be performed during DBE. In addition, a squeeze sign has been described to diagnose lipomas, using changes in the outline and size during peristalsis and pressure on fluoroscopy [12]. EGD and CS are not optimal methods for detection because of the deep position, anatomical tortuosity, and endoscopic inaccessibility of small intestinal lipomas. CE and DBE have increased the rate of diagnosis of small intestinal tumors [13]. Although CE enables visualization of the entire small intestine, this new technique has limitations, including contraindications, difficulties in identifying the location of abnormal findings, and a lack of tissue sampling as well as therapeutic properties. In contrast, DBE possesses diagnostic and therapeutic advantages over CE.

Although the majority of patients are asymptomatic, it is necessary to treat GI lipomas if they cause symptoms. Previous studies suggest that endoscopic snare removal of small lipomas (<2 cm) is safe and efficacious [14]. However, the outcome of endoscopic removal of a large lipomas (>2 cm) is not well defined, because the risk of perforation and severe hemorrhage increases with lipoma size and border base [14]. Some studies indicate that large GI lipomas can be removed safely using endoscopic treatment [4, 6]. Endoscopic treatment may be applied in various ways: the unroofing technique, polypectomy, endoscopic mucosal resection (EMR), or endoscopic submucosal dissection (ESD). The unroofing technique can be used to extract large GI lipomas, but there is potential for recurrence using this technique. EMR is used predominantly for small lipomas (<2 cm) [6]. In the resection of large lipomas, the size and shape of the stalk is considered to be more important than the size of the lipoma itself [15]. In cases in which it can be difficult to resect by EMR alone, ESD may be considered. However, as in the present case, endoscopic treatment was not recommended because the endoscopic treatment was difficult and risky. In addition, large lipomas may have ulcerations as a result of pressure necrosis from the overlying mucosa, and large lipomas cause GI bleeding and anemia [16, 17]. In the previous reports, some OGIB cases with lipoma also underwent antiplatelet therapy. Therefore, antiplatelet therapy may be a risk factor for OGIB [9, 10]. We speculated that a patient with a large ulcerated lipoma who was undergoing antiplatelet therapy would have a high risk of bleeding. In such cases, surgery is the feasible therapy for the majority of symptomatic patients, including explorative laparotomy and laparoscopic-assisted resection [9].

## Conclusions

Small intestinal lipomas are rare, but GI bleeding represents an opportunity for the discovery of large lipomas in patients on antiplatelet therapy. CE and DBE are good modalities for the diagnosis of small intestinal lipomas. Endoscopic treatment in the small intestine should be performed carefully, even if the endoscope can reach the lipoma site.

Surgical resection may be a feasible and safe therapy for most symptomatic lipomas. Treatment of small intestinal lipomas should be selected carefully, considering the size of lipoma, size of stalk, administration of antithrombotic therapy, and endoscopic operability.
